# Multifocal osteochondromatous proliferation and paraneoplastic hematologic dyscrasia in the context of latent Epstein-Barr virus reactivation: a case of oncologic and infectious pathophysiology

**DOI:** 10.1007/s00256-025-04872-y

**Published:** 2025-01-17

**Authors:** Zeyad Hossam Atta Khalil, Taha Ali Osman

**Affiliations:** College of Medicine/Radiology Department, October 6, University, 217G Pyramid Gardens, Cairo, Egypt

**Keywords:** Epstein-Barr virus (EBV), Multifocal osteochondromatous proliferation, Paraneoplastic hematologic dyscrasia, Bone marrow dysplasia

## Abstract

This case report describes a 15-year-old male with multifocal osteochondromatous proliferation and paraneoplastic hematologic dyscrasia, linked to latent Epstein-Barr virus reactivation. Radiographic and advanced imaging revealed widespread skeletal lesions consistent with osteochondromatosis. Hematologic evaluation indicated pancytopenia with dysplastic megakaryocytes and marrow infiltration. Immunohistochemical staining confirmed latent Epstein-Barr virus infection, suggesting its role in the pathogenesis of both the osteochondromatous and hematologic abnormalities. This case highlights the correlation between Epstein-Barr virus reactivation, bone proliferation, and paraneoplastic hematologic processes, which we believe has not yet been reported in the literature, emphasizing the need for a comprehensive diagnostic approach.

## Introduction

Multifocal osteochondromatous proliferation is an exceedingly rare skeletal disorder characterized by the development of multiple osteochondromas across various anatomical sites [1]. Unlike hereditary multiple exostoses, which is associated with mutations in the EXT1 and EXT2 genes and typically presents with multiple bony outgrowths during childhood, multifocal osteochondromatous proliferation can arise sporadically without a known genetic predisposition [2]. These lesions consist of both bone and cartilage, usually exhibiting continuity with the underlying bone cortex and medullary cavity; nevertheless, discontinuity was observed in this case which is atypical [3]. The proliferative nature of these lesions can result in complications such as skeletal deformities, impingement on adjacent structures, restricted joint movement, and, in some cases, malignant transformation to chondrosarcoma [4]. The clinical course of multifocal osteochondromatous proliferation is variable, ranging from asymptomatic presentations to severe skeletal morbidity [5].

Paraneoplastic hematologic dyscrasias, on the other hand, encompass a spectrum of hematologic abnormalities that arise secondary to an underlying neoplastic process, often without direct invasion of the bone marrow by the primary tumor [6]. These dyscrasias can manifest as anemia, thrombocytopenia, leukocytosis, or a more complex pancytopenia [7]. They are often driven by the production of cytokines, autoantibodies, or other factors released by the neoplasm that disrupt normal hematopoiesis [8]. In pediatric patients, paraneoplastic syndromes are more commonly associated with solid tumors, such as neuroblastoma or Wilms’ tumor, and rarely with benign bone conditions, making this an atypical and challenging diagnosis [9].

Epstein-Barr virus (EBV), a member of the herpesvirus family, is widely recognized for its role in various lymphoproliferative diseases and its oncogenic potential [10]. EBV infects over 90% of the global population, establishing latency primarily in B lymphocytes [11]. While primary infection is typically asymptomatic or presents as infectious mononucleosis, latent infection can persist for the host’s lifetime [12]. Reactivation of latent EBV has been implicated in the pathogenesis of several conditions, including nasopharyngeal carcinoma, Hodgkin’s lymphoma, and post-transplant lymphoproliferative disorders [13]. However, EBV’s association with non-malignant, proliferative bone conditions remains largely unexplored, particularly its role in modulating the bone microenvironment in multifocal osteochondromatous proliferation [14].

This report focuses on a case of multifocal osteochondromatous proliferation co-existing with paraneoplastic hematologic dyscrasia. The rarity of such a presentation, especially in the absence of an underlying malignancy, and its potential link with latent EBV reactivation, highlights the need for a deeper exploration into the underlying mechanisms and clinical implications of this confluence of disorders.

## Case report

A 15-year-old male presented with progressive skeletal deformities, noticeable bony masses, and intermittent joint discomfort. Over the past year, he experienced increasing difficulty in ambulation due to pain and stiffness, predominantly affecting the lower extremities. Additionally, he reported fatigue, unexplained weight loss, and episodic low-grade fevers over the last 6 months. There was no significant family history of skeletal disorders or malignancies, and the patient’s past medical history was unremarkable, without prior trauma or infection.

On physical examination, multiple firm, non-tender osseous masses were palpable, particularly across the metaphyseal regions of the femur, tibia, and vertebrae. A mild valgus deformity was observed in the left knee, along with restricted range of motion in both the hip and knee joints. Neurological examination was normal, with no evidence of hepatosplenomegaly, lymphadenopathy, or cutaneous abnormalities.

Laboratory investigations revealed significant pancytopenia, with a hemoglobin level of 8.2 g/dL, white blood cell count of 2.5 × 10^9^/L, and platelet count of 75 × 10^9^/L. Serum lactate dehydrogenase (LDH) was markedly elevated at 650 U/L, and ferritin levels exceeded 1000 ng/mL, suggestive of an inflammatory or neoplastic process. Peripheral blood smear showed dysplastic changes in the myeloid lineage, with no circulating blasts. Coagulation profiles and autoimmune screening, including ANA and anti-dsDNA antibodies, were within normal limits.

**Fig. 1:**
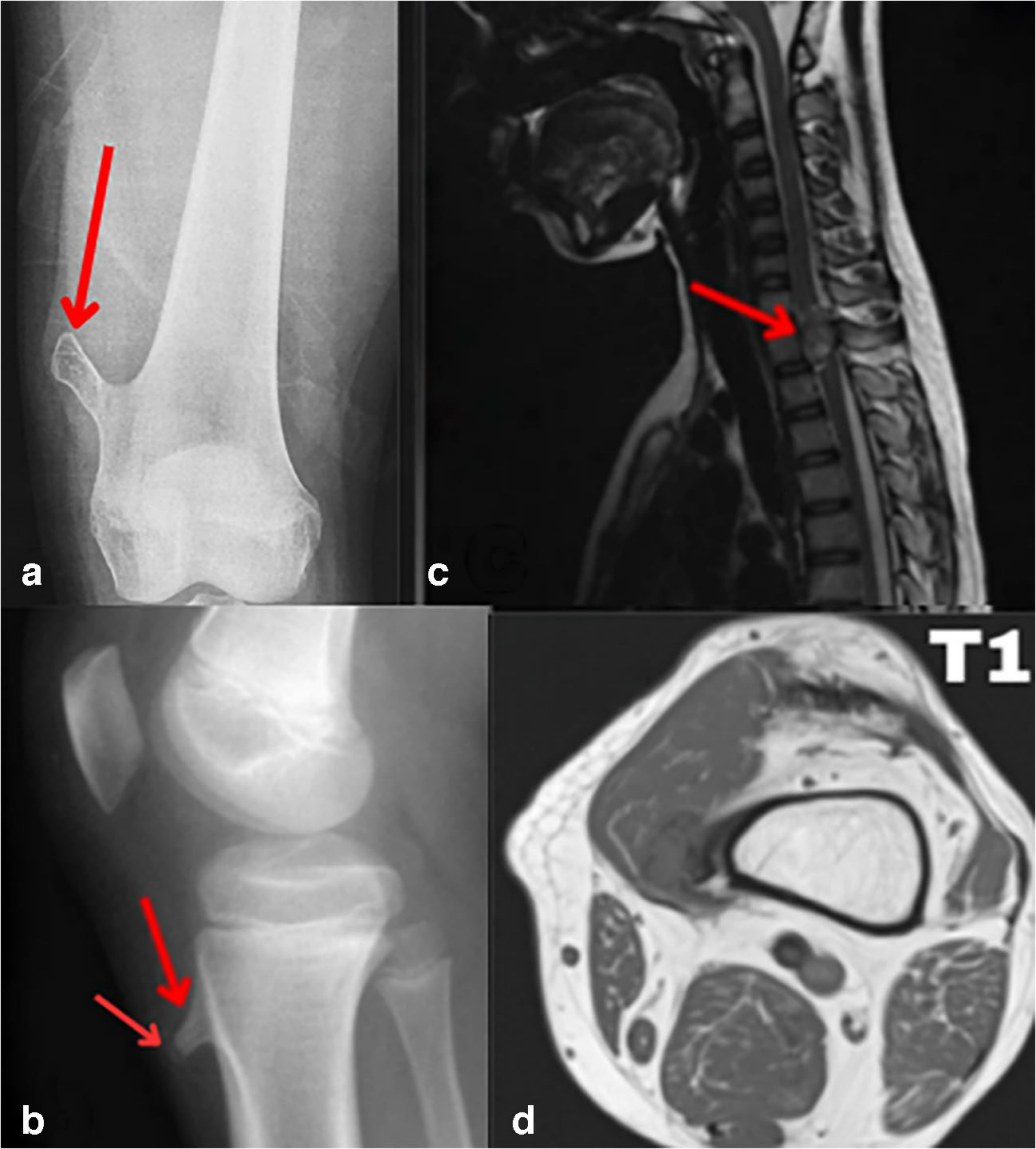
The AP radiograph image of the knee (**a**) shows a well-defined lesion in the medial aspect of the femoral metaphyses directed away from the joint. The knee joint is normal in appearance. The lesion was hard in nature and found as a lump in the left thigh with the thought of potential continuity, which was disapproved, due to the absence of clear continuity on advanced imaging. The lateral radiograph image (**b**) reveals a sessile ossific tibial metaphyseal lesion and a small bony protrusion from the anterior metaphysis, separated from the growth plate, with chondroid calcification and no continuity with the adjacent bone marrow, which was initially thought as well to be continuous but appeared on advanced imaging otherwise. A second arrow points to the chondroid calcification found at the anterior margin of the lesion. A STIR MR of the cervical spine in image (**c**) demonstrates a well-defined lesion extending into the spinal canal, causing mass effect and early myelopathic changes in the dorsal spinal cord; STIR MRI was performed specifically in this case due to its capability to suppress fat signals, which was essential for visualizing the osteochondromatous lesion without interference from overlapping fat signals. Posterior scalloping of the T2 vertebral body is also evident, highlighting the lesion’s pressure impact and remodeling effects. The T1-weighted MRI shown here in (**d**) demonstrates the lack of continuity of the lesion in the distal femur with the medulla. The lesion’s low intensity on the T1 image is also evident

**Fig. 2:**
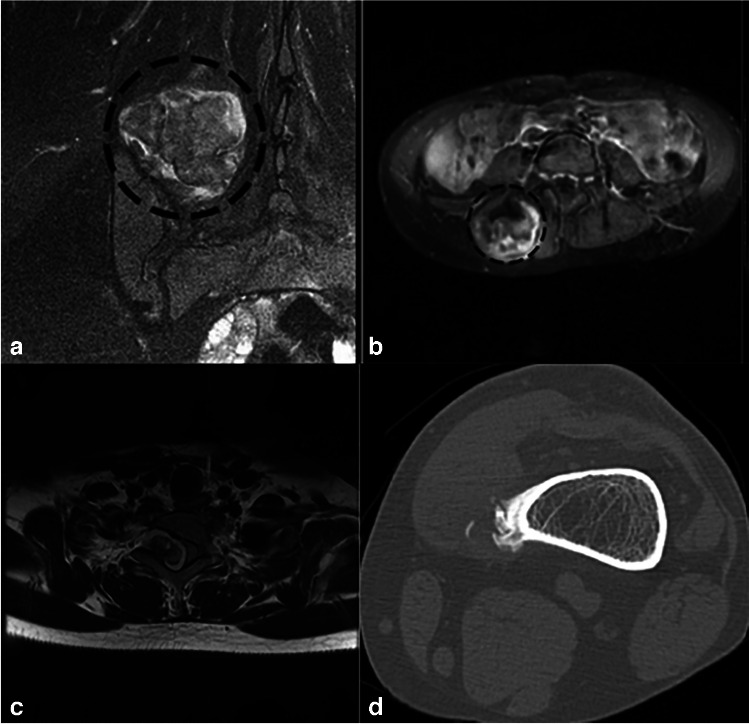
The coronal and axial T2 FS post-contrast. Images (**a**) and (**b**) show a large, multiloculated osteochondroma arising from the posterior right iliac crest, extending slightly into the superior sacroiliac joint and laterally toward the right paraspinal muscles and is non-continuous. Post-contrast images show patchy enhancement in the posterior osseous component, though its significance is unclear. No fractures or bursa formations are identified, and there is no associated soft tissue mass. Degenerative changes in the right sacroiliac joint include small subchondral cysts. The pelvis appears to be clear of masses. Image (**c**) shows an axial T2 MRI detailing the exact location of the T2 lesion relative to the vertebral body. A pedunculated bony outgrowth originates from the right superior articular process of the T2 vertebra. This lesion presents with a T2 hypointense stalk and a T2 hyperintense signal in its cap. Post-contrast imaging reveals heterogeneous enhancement of the lesion, which extends into the spinal canal, resulting in myelopathic changes in the adjacent dorsal spinal cord. Image (**d**) illustrates a CT scan displaying a widespread ossified mass adjacent to the medial cortex of the distal femur, with no continuity observed between the mass and the medullary cavity

Radiographic imaging in Figs. 1 and 2 of the skeletal system showed multiple, well-circumscribed, pedunculated, and sessile osseous lesions which showed potential cortical and medullary continuity at first but was revealed by advanced imaging to be non-continuous as shown in Fig. 1D and as shown in the x-ray in Fig. 1C. Computed tomography of the pelvis and lower limbs revealed extensive cartilaginous caps, with several lesions displaying heterogeneously calcified cores, measuring up to 4 cm in diameter. Microscopically, the cartilage cap exhibited low cellular density, with chondrocytes distributed evenly throughout the matrix and without evidence of nuclear atypia or pleomorphism. This absence of cellular irregularity is critical, as malignant transformation in osteochondromas, such as progression to chondrosarcoma, typically shows disorganized, hypercellular regions with irregularly shaped and atypical nuclei. The cartilage also lacked zones of abnormal mitotic activity, a hallmark of malignant growth. The cap was also notably free of binucleated cells, often observed in malignant cartilage. The smooth transition between the cartilage cap and underlying bony stalk further indicated a lack of invasive potential, as malignancies tend to disrupt this boundary. The medullary cavity beneath the lesions retained a typical pattern of bone marrow elements, with no evidence of osteolytic activity or periosteal reaction, frequently associated with malignancy. Magnetic resonance imaging demonstrated high signal intensity on T2-weighted images and low signal on T1-weighted images in the cartilaginous regions, without evidence of malignant transformation. Notably, the lesions showed no connection with the medullary cavity, distinguishing them from classical osteochondromas (Fig. 1D).

Further evaluation with a bone marrow biopsy was performed, revealing hypocellular marrow with an atypical proliferation of dysplastic megakaryocytes, immature myeloid precursors, and extensive macrophage activation. The marrow showed no evidence of leukemia, lymphoma, or myelodysplasia, but there was an absence of normal hematopoiesis. Immunohistochemical staining of the marrow specimens demonstrated positivity for CD68 in macrophages, while CD34 and CD117 staining in the myeloid series were abnormal, indicating a paraneoplastic hematologic process.

**Fig. 3:**
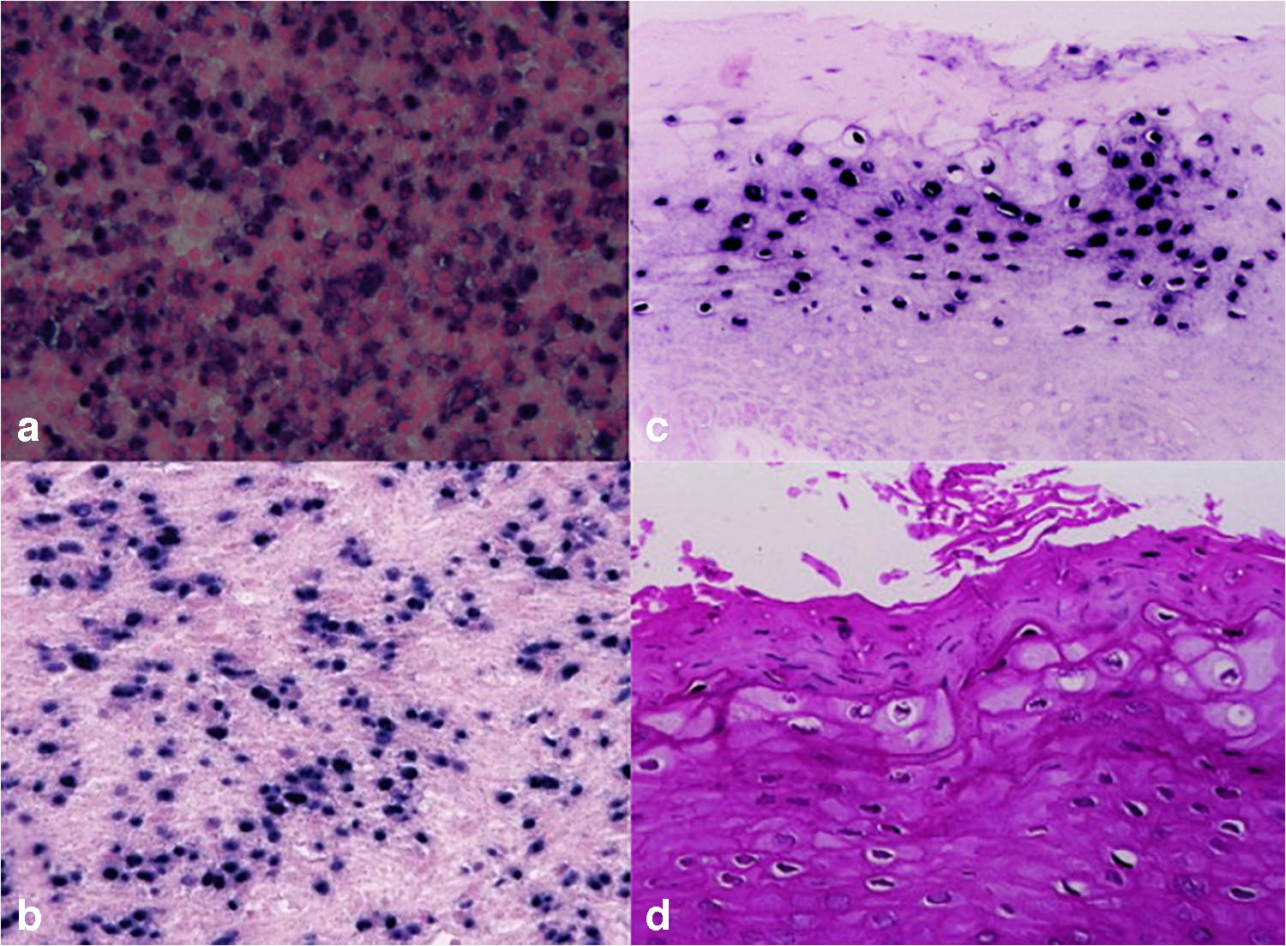
In situ hybridization for Epstein-Barr virus (EBV) from bone marrow biopsy of femoral metaphyseal lesion. **a** Bone marrow smear showing cellularity. **b** Positive EBER staining in bone marrow cells, indicated by dark nuclear signals, confirming EBV presence. **c** Higher magnification highlighting nuclear EBER positivity in hematopoietic cells. **d** ISH staining of the osteochondromatous lesion showing nuclear EBER signals in stromal and chondrocytic cells, indicating EBV’s involvement in both the hematologic and skeletal abnormalities

Given the unusual presentation, additional testing for viral etiologies was conducted. Quantitative polymerase chain reaction of the blood and marrow tissue identified a high viral load of Epstein-Barr virus (EBV) DNA. In situ hybridization for EBV-encoded RNA in the bone marrow biopsy was positive (Fig. 3), confirming the presence of latent EBV infection within the hematopoietic and stromal cells. This finding suggested a direct involvement of EBV in the pathogenesis of the patient’s hematologic abnormalities and possibly in the osteochondromatous proliferation.

**Fig. 4:**
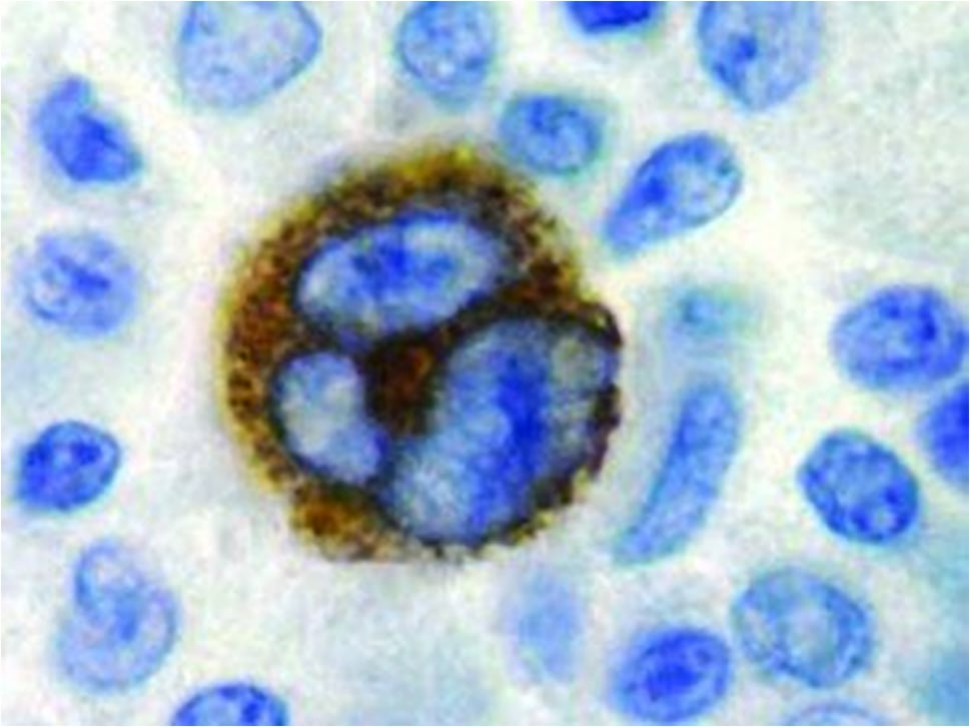
Immunohistochemical staining of the excised osteochondromatous lesion, showing strong nuclear and cytoplasmic positivity for Epstein-Barr virus (EBV) latent membrane protein 1 within a chondrocytic cell. The presence of latent membrane protein 1, marked by the brown staining, indicates active EBV involvement within the lesion’s cellular matrix. Surrounding cells exhibit typical morphology without latent membrane protein 1 expression, highlighting the localized EBV infection in the osteochondromatous tissue

High-resolution imaging of the skeletal lesions was performed, and a representative lesion from the femoral metaphysis was surgically excised for histopathological analysis. Gross examination of the excised mass revealed a lobulated, firm growth with a cartilaginous cap and underlying osseous tissue. Histology showed an outer layer of disorganized cartilage with areas of endochondral ossification and a central core of mature trabecular bone, resembling osteochondromatous proliferation. Immunohistochemistry of the lesion confirmed the presence of chondrocytes expressing S100 protein, without evidence of malignant features such as nuclear atypia or increased mitotic activity. Notably, the stromal components of the lesion were positive for EBV latent membrane protein 1 (Fig. 4), implicating EBV in the etiology of the multifocal osteochondromatous process.

The patient’s condition was managed with a multidisciplinary approach. Supportive hematologic care included transfusions and immunomodulatory therapy aimed at controlling the paraneoplastic hematologic dyscrasia. Given the association with latent EBV, antiviral therapy with valganciclovir was initiated. Orthopedic intervention was limited to symptomatic lesions, with close monitoring for potential malignant transformation. Over a 6-month follow-up period, the patient showed partial hematologic improvement and stabilization of the skeletal lesions, though persistent low-grade viremia indicated ongoing EBV activity.

## Discussion

Hereditary multiple exostoses (HME) was a primary consideration due to the presentation of multiple osteochondromas. The patient’s age at onset and lack of progressive growth patterns characteristic of HME-associated lesions suggested a non-hereditary etiology. Collectively, these findings, absence of family history, negative genetic testing, atypical lesion distribution that usually includes lesions found in the hip, and shoulder, which is atypical here, and unique clinical progression effectively ruled out HME as a diagnosis.

Metastatic disease was also considered, especially since multifocal bone lesions may mimic metastatic spread, particularly in cancers with a known propensity for bone involvement, such as breast, prostate, and lung cancers. However, advanced imaging revealed the discontinuity of the lesion’s medullary cavities with adjacent bone, which is a mark for osteochondromas rather than metastatic deposits, which usually present with lytic, destructive features. Additionally, there was no history of primary malignancy nor evidence of visceral or other systemic involvement in further imaging studies.

Multiple myeloma often presents with multifocal lytic bone lesions and a complex paraneoplastic hematologic profile; however, serum protein electrophoresis and bone marrow biopsies demonstrated no monoclonal protein spike or plasma cell infiltration characteristic of myeloma. Lymphoma was similarly ruled out, as bone marrow analysis lacked the lymphoid aggregates or abnormal cellular morphology expected in hematologic malignancies. These factors collectively supported the conclusion of EBV-driven pathogenesis involving bone lesions, aligning with the rare but increasingly recognized capability of EBV to impact bone and marrow homeostasis.

EBV’s ability to infect a broad range of cell types, including mesenchymal cells, aligns with the findings in this case, where the virus’s presence in both the bone marrow and osteochondromatous lesions suggests a multifaceted pathogenic role. The precise pathways through which EBV contributes to such a complex clinical presentation remain speculative, but they likely involve the virus’s manipulation of the host’s cellular machinery. EBV is known to express proteins such as LMP1 and LMP2, which can activate a variety of intracellular signaling cascades, including NF-κB, JAK/STAT, and PI3K/Akt pathways, promoting cellular proliferation and survival [15]. Latent membrane protein 1, in particular, is capable of inducing the expression of RANKL in the bone microenvironment, which can lead to an imbalance in bone remodeling by enhancing osteoclast activity and potentially driving the formation of osteochondromas [16]. This aberrant signaling may have contributed to the multifocal nature of the osteochondromatous proliferation observed in this patient.

Furthermore, EBV’s interaction with the bone marrow environment could explain the patient’s paraneoplastic hematologic dyscrasia. The virus’s infiltration of hematopoietic and stromal cells may have altered the cytokine milieu, promoting a pro-inflammatory state. The release of cytokines such as IL-6 and TNF-α not only disrupts normal hematopoiesis but also induces an immune response that can damage the bone marrow [17]. This inflammatory microenvironment can facilitate the expansion of dysplastic megakaryocytes and myeloid precursors, resulting in the observed pancytopenia. The correlation between EBV and paraneoplastic syndromes has been noted in other contexts, such as in the association between EBV-positive lymphomas and hemophagocytic lymphohistiocytosis (HLH), where EBV triggers a hyperinflammatory response [18]. While HLH was not diagnosed in this case, the underlying mechanism of immune activation and marrow infiltration may share similarities.

In addition, our hypothesis for the benign nature of the thick cartilage caps found in the case could relate to a unique interaction between the Epstein-Barr virus (EBV) and the patient’s mesenchymal cells within the osteochondromatous lesions. EBV has been shown to induce alterations in cell signaling pathways such as NF-κB and PI3K/Akt, which can promote proliferation and cell survival without necessarily inducing malignant transformation. In this case, the presence of EBV in both bone marrow and lesion tissues, as confirmed by in situ hybridization, may have contributed to increased cartilage proliferation within the caps, without activating the full spectrum of oncogenic mechanisms required for malignancy.

This case also raises the possibility that the paraneoplastic-like syndrome observed here is not merely a secondary effect of the osteochondromatous lesions but rather part of a unified process driven by EBV. The virus’s ability to establish latency and periodically reactivate suggests a cyclical influence on both bone and hematopoietic tissue. Reactivation could lead to episodic inflammatory and proliferative surges, contributing to the dynamic nature of the patient’s clinical course. The presence of EBV within the marrow and the osteochondromatous lesions suggests that the virus might create a local microenvironment that supports both dysplastic hematopoiesis and abnormal bone growth. This dual influence underscores the importance of considering viral factors in the etiology of atypical paraneoplastic syndromes and multifocal bone proliferative disorders.

In terms of therapeutic implications, the identification of EBV as a potential driver of these pathologies suggests that antiviral therapy might play a role in managing similar cases. While antivirals like ganciclovir and valganciclovir have shown efficacy in reducing EBV viral load in certain conditions, their role in modifying the course of EBV-associated bone and hematologic disorders remains uncertain [19]. Furthermore, immunomodulatory therapies that target the inflammatory response, such as corticosteroids or cytokine inhibitors, may be considered to mitigate the effects of the virus-induced immune dysregulation. This case emphasizes the need for a multidisciplinary approach that combines antiviral, hematologic, and orthopedic interventions, tailored to address the multifaceted nature of the disease.

The relation between viral infection and host cellular processes in this case is complex and highlights the need for further research into the mechanisms of EBV-related bone and hematologic pathologies. It remains unclear why, in some patients, EBV infection results in benign proliferative lesions like those seen here, whereas in others, it leads to malignancy. Also, why it can show medullary continuity in one patient and the absence in the other? Genetic predisposition, host immune status, and viral load may all contribute to the clinical variability observed in EBV-associated conditions. Additionally, the possibility of other co-infections or environmental factors influencing the course of the disease cannot be excluded. This case illustrates the intricate relation between EBV reactivation, multifocal osteochondromatous proliferation, and paraneoplastic hematologic dyscrasia. It presents a potential novel hypothesis wherein EBV acts as a driver of both skeletal and hematologic pathologies, emphasizing the need for comprehensive diagnostic evaluations in similar presentations.

## Data Availability

Data supporting this study are available upon reasonable request by contacting the corresponding author. Access to the data is subject to compliance with patient confidentiality and privacy regulations.
